# Spontaneous regression of Merkel cell carcinoma in a patient with chronic lymphocytic leukemia: a case report

**DOI:** 10.1186/1752-1947-3-7270

**Published:** 2009-05-27

**Authors:** Tamara Turk, Zeljka Crncevic Orlic, Ivana Smoljan, Antica Nacinovic, Irena Seili Bekafigo, Jelena Radic, Gordana Zamolo

**Affiliations:** 1 Department of Internal Medicine, School of Medicine, University of Rijeka, Br. Branchetta 20, Rijeka, Croatia; 2 Department of Internal Medicine, University Hospital Rijeka, Cambierieva 17/5, Rijeka, Croatia; 3 Department of Pathology, School of Medicine, University of Rijeka, Br. Branchetta 20, Rijeka, Croatia

## Abstract

**Introduction:**

Merkel cell carcinoma is a rare and aggressive primary cutaneous neuroendocrine malignant tumor. The tumor has a high rate of local recurrence after surgical removal. Spontaneous regression appears to be relatively common in this rare type of tumor.

**Case presentation:**

We describe the clinical course, cytological and histological findings of a Merkel cell carcinoma in a 70-year-old Caucasian woman, simultaneously diagnosed with chronic lymphatic leukemia. The tumor showed clinical regression after fine needle aspiration. At primary presentation, the tumor had no apparent leukocyte infiltration, but was completely cleared by T-cell mediated immunity within 3 weeks after fine needle aspiration.

**Conclusion:**

Fine needle aspiration may have acted as a mechanical trigger involved in the activation of cell-mediated immunity, leading to the clinical and histological regression of the tumor. To the best of our knowledge, this is the first case report of spontaneous regression of Merkel cell carcinoma in a patient with a co-malignancy, that is to say, chronic lymphocytic leukemia.

## Introduction

Merkel cell carcinoma (MCC) is a rare, aggressive skin tumor known for its high rate of recurrence, metastasis formation and consequently, mortality [1]. It is a primary cutaneous neuroendocrine malignant tumor [2] and commonly occurs in sun-exposed areas of the body, mostly in the head and neck region (65%), even though the upper extremities are involved in 18% and the lower extremities in 13% of cases [3]. The clinical presentation of MCC is usually painless, firm, erythematous, intradermal or subcutaneous nodules without ulceration [4]. According to the literature, co-malignancies are associated with a higher rate of mortality. The most common ones are other types of skin tumors and hematological malignancies [5]. Although MCC is an aggressive neoplasm, spontaneous regression has been reported in several cases, with an estimated prevalence of 1.7% to 3% [6,7].

We describe the clinical course, cytological and histological findings of MCC in a 70-year-old woman, simultaneously diagnosed with chronic lymphatic leukemia (CLL). The tumor has shown clinical regression after fine needle aspiration (FNA). To the best of our knowledge, this is the first case report of spontaneous regression of MCC in a patient with hematological co-malignancy, although spontaneous regression of MCC has been reported earlier in 89-year-old man with a solid co-malignancy of lung adenocarcinoma [8].

## Case presentation

**Figure 1:**
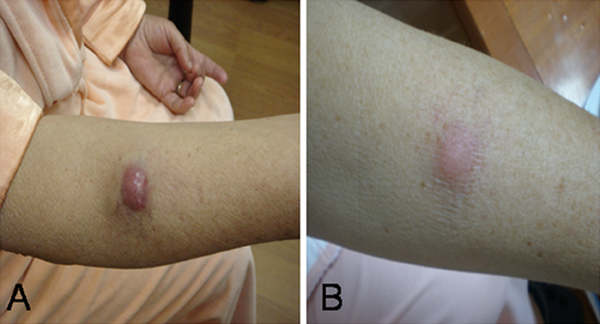
**Clinical presentation of tumor**. **(A)** 70-year-old Caucasian woman with a rapidly growing tumor on her right forearm, which had appeared 2 months earlier, allegedly after an unidentified insect bite. **(B)** Spontaneous clinical regression of the tumor.

We present a 70-year-old Caucasian woman with a rapidly growing tumor on her right forearm (Figure 1A), which had appeared 2 months earlier, allegedly after an unidentified insect bite. A few weeks later, she noticed a small red nodule in that area. During the next few weeks, the nodule started growing rapidly so she was referred to our hospital. She had no other complaints. Physical examination revealed a solitary, firm, non-ulcerated, livid-red nodule 3 cm in diameter. The regional lymph nodes were not enlarged. Routine hematology tests revealed an elevated white blood cell (WBC) count (17 × 10^9^/L) with 75% lymphocytes, suggesting a lymphoproliferative disorder.

**Figure 2:**
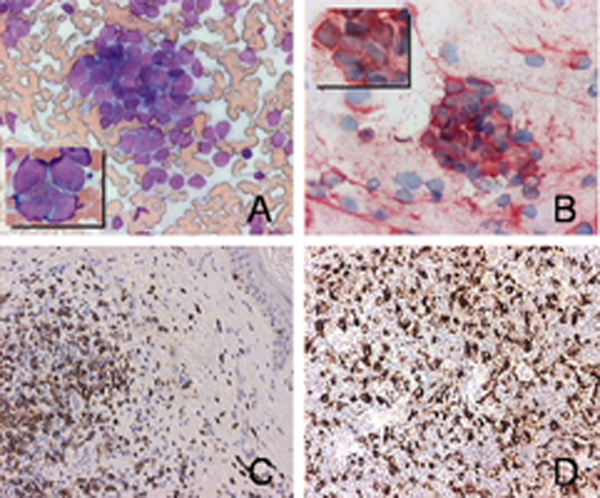
**Cytopathological examination of the tumor and regression lesion**. **(A)** Fine needle aspiration - abundant cellularity, clusters of atypical cells without visible nucleoli, prominent nuclear molding (May-Gruenwald-Giemsa ×200, insert ×1000). **(B)** Immunostaining for Ber-Ep4 (×200, insert ×1000). **(C)** Immunohistochemical labeling of infiltrating lymphocytes showed a dense infiltrate of CD3+ cells (pan-T cell) (×400). **(D)** Immunohistochemical labeling showed a dense infiltrate of CD68+ cells (monocytes/macrophages) (×400).

Fine needle aspiration (FNA) was performed in two separate areas of the nodule and smears were stained in accordance with the May-Gruenwald-Giemsa method. Both specimens showed an abundance of relatively monomorphic, atypical, epithelial-appearing cells without visible nucleoli, lying alone and in clusters. Occasional mitoses were evident. There were a few lymphoid cells in the background, very scant fibrous stroma and some peripheral blood. Nuclear molding was prominent in these cells and the cytoplasm was scant and rarely preserved (Figure 2A). Malignant cells showed strong staining with epithelial antigen (Ber-Ep4) (Figure 2B), while leukocyte common antigen (LCA) was used for leukocytes. Malignant cells showed strong positivity for epithelial marker, while LCA marked only a few scattered lymphoid cells in the background. Diagnosis of epithelial malignant tumor with neuroendocrine morphologic features indicated Merkel cell carcinoma.

Simultaneously with the FNA of the cutaneous tumor, a peripheral blood smear was analyzed. This showed a lymphocytosis (75% of lymphocytes). Bone marrow aspiration and flow cytometric analyses confirmed the diagnosis of a low-grade B-cell chronic lymphocytic leukemia. Right axillary region ultrasound indicated two enlarged lymph nodes which showed reactive changes on histopathological examinations. Further diagnostic tests did not reveal lymphadenopathy, hepatomegaly or splenomegaly. Whole body skeletal scintigraphy imaging did not indicate any irregularities. In our patient, CLL was in Rai stage 0, so there was no need for active treatment.

During 3 weeks of hospital treatment, we noticed the spontaneous regression of the tumor to half of its original size (Figure 1B). Surgical excision and sentinel lymph node biopsy were then carried out. Radical excision with 2 cm of tumor-free margins was performed under general anesthesia. The sentinel lymph nodes did not contain MCC.

**Figure 3:**
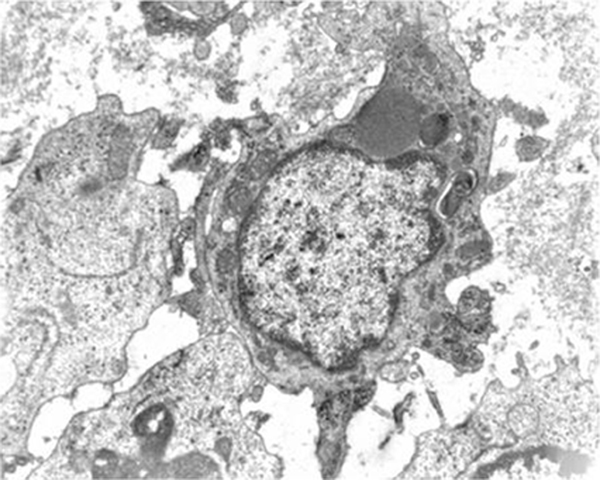
**Electronic microscopy of the excised tumor**. Histiocytic lesion: giant cell with irregular cytoplasmic sprouts. Erythrophagocytes with large mitochondria, lysosomes, rough endoplasmatic reticulum and some lipid drops were found in the cytoplasm. Electron microscopy: ×7100.

Histopathological analysis of the regressed tumor, stained with hematoxylin and eosin, revealed that the tumor cells were completely replaced by mononuclear cells. An immunohistochemical study was performed with formalin-fixed and paraffin embedded material, using the avidin-biotin peroxidase method (all antibodies from Dako Japan, Co. Ltd, Kyoto). Immunolabeling of infiltrating lymphocytes was positive for CD3+ T cells (Figure 2C), while there were a few B cells. Infiltrating macrophages were positive for CD68+ (Figure 2D). Electronic microscopy of the excised tumor showed histiocytic infiltration, with no trace of epithelial cells (Figure 3).

The patient was discharged from hospital 7 days after the surgery and was referred to local radiotherapy. She was re-admitted to our hospital for an evaluation 6 months after the initial presentation. Her CLL was stable. Results of an FNA around the excision site showed no recurrence of the tumor and no lymph node involvement was noticed.

## Discussion

This case report represents the first case of a spontaneous regression of MCC in a patient with a hematological co-malignancy, CLL. So far, several studies have reported the appearance of MCC in patients with CLL [9]-[12], but none with MCC as the first presentation. Furthermore, in all of the previously reported cases of spontaneous regression, the tumor was localized in the head and neck region [6]-[13], while in our example, the tumor site was on the right forearm.

The incidence of MCC is high (8%) among immunosuppressed patients [2], but the pathogenetic relationship between CLL and cutaneous neoplasia is still unclear. Both UV radiation and CLL are thought to cause immunosuppression which is associated with an increased incidence of cutaneous malignancies.

In our patient, these factors were combined with another possible trigger - an insect bite, generating an inflammatory milieu from which MCC could have derived. The fact that the tumor developed a few weeks after the insect bite, favors this line of thought.

A better prognosis has been associated with shorter intervals between the appearance of CLL and development of MCC, even though the outcome of MCC is possibly independent of the CLL stage [14]. Poor prognostic factors are young age, male sex, large tumor size, truncal site, nodal or distant disease and duration of the disease before presentation [2,15]. Considering these parameters, a favorable outcome would be expected in our patient.

Regression is a complete or partial disappearance of malignant cells without any treatment. It is mediated by T-lymphoid infiltration which induces apoptotic tumor cells [16]. A high proportion of MCC shows regression (1.7 to 3%) in MCC patients [17] so immunologic responses play an important role in this type of tumor.

It is still unclear how a mechanical stimulus (FNA) can be a trigger for activating the immune system toward macrophage mediated digestion of malignant cells. All previously described cases of MCC regression without any treatment were also noticed after needle biopsy. This procedure could stimulate the immune system toward tumor destruction. Our findings indicate that FNA is responsible for the activation of cell-mediated immunity, leading to clinical and histological regression of the tumor.

Results of our histological examination showed that the tumor cells were completely replaced by mononuclear cells. Immunohistological staining showed that the majority of cells were CD3+ T-lymphocytes and CD 68+ macrophages, while there were a few B-lymphocytes. Electronic microscopy revealed clusters of histiocytes containing phagocytosed tumor cells. Our pathohistological findings point to the fact that spontaneous regression is due to the T-cell mediated immunity, which is the case with our patient who suffers from an early stage B cell leukemia. This is consistent with the work of Maruo *et al.*, who suggested that T-cell immunity plays an important role in spontaneous regression of MCC. In their case, tumor cells were also replaced by macrophages [13].

## Conclusion

We should draw attention to the possibility of fine needle aspiration being a trigger of regression. This suggests that Merkel cell carcinoma is highly responsive to immunologic reactions, so a possible next step to consider in its treatment could be immunotherapy.

At this point, many questions still remain unanswered and additional case reports are necessary to shed light on the mechanisms of spontaneous regression of MCC.

## Abbreviations

MCC: Merkel cell carcinoma; CLL: chronic lymphatic leukemia; WBC: white blood cells; FNA: fine needle aspiration; LCA: leukocyte common antigen; Ber-Ep4: epithelial antigen.

## Consent

Written informed consent was obtained from the patient for publication of this case report and any accompanying images. A copy of the written consent is available for review by the Editor-in-Chief of this journal.

## Competing interests

The authors declare that they have no competing interests.

## Authors' contributions

TT carried out the hematological examination and participated in the design of the study. ZCO, IS and AN carried out the hematological examination and participated in coordination and drafting of the manuscript. IS performed the cytological examination and participated in writing of the manuscript. JR and GZ carried out the pathohistological and immunohistochemical examination and participated in writing of the manuscript. All authors read and approved the final manuscript.
